# Variations of the Cystohepatic Blood Supply in American Midwestern Donor Cadavers

**DOI:** 10.7759/cureus.32260

**Published:** 2022-12-06

**Authors:** Shayan A Memar, Caroline VanSickle, Sara Funk, Jeremy J Houser, Shanu Markand

**Affiliations:** 1 Anatomy, A.T. Still University of Health Sciences - Kirksville College of Osteopathic Medicine, Kirksville, USA

**Keywords:** anatomical variation, cystic triangle, hepatobiliary system, calot’s triangle, cystic artery, right hepatic artery

## Abstract

Knowledge of right hepatic artery (RHA) and cystic artery (CA) variations is crucial for surgeons performing procedures on the hepatobiliary system, pancreas, and duodenum. Commonly, the RHA originates from the superior mesenteric artery (SMA), while the CA originates from the RHA and is found within the cystic triangle during laparoscopic cholecystectomies. Here we investigated variations in the origin and path of the RHA and CA in a sample of American midwestern cadavers (n = 18) from the Gift of Body Program at A.T. Still University’s Kirksville College of Osteopathic Medicine. Portal triads and associated vessels were dissected to reveal the artery pathways. The origin, branching pattern, and course of the RHA and CA were documented, and descriptive measurements were taken. We describe four cases where the RHA originated from the anterolateral proximal SMA, traveled deep to the pancreatic neck, and had a slightly variable but close relationship with the portal triad structures. The CA was present in the cystic triangle in all 18 donors, typically originating from the RHA except for one case where it originated from the left hepatic artery. In six cases, the CA originated outside of the cystic triangle, crossing either superficially or deeply to the common hepatic duct to enter the cystic triangle. Knowledge of these variations will enhance preoperative planning and the overall safety of surgical procedures in this area.

## Introduction

The celiac trunk, superior mesenteric artery (SMA), and inferior mesenteric artery are the primary parent vessels that supply the gastrointestinal system. Due to the complex nature of their embryological development, anatomical variations are commonly found in the origin and course of the vascular derivatives of the celiac trunk and SMA [1-4]. Knowledge of these variations is clinically pertinent for surgical procedures, diagnostic studies, and radiological interventions [3,5-11]. During common procedures such as cholecystectomies or pancreaticoduodenectomies, it is imperative that surgeons understand the possible vascular variations to ensure safe patient outcomes.

Anatomy of the Celiac Trunk and Superior Mesenteric Artery

The celiac trunk arises from the ventral surface of the abdominal aorta as the first major unpaired vessel that provides branches that include the common hepatic artery. The common hepatic artery typically provides branches supplying the liver, gallbladder, pancreas, and proximal duodenum [12]. The common hepatic artery terminates by dividing into the gastroduodenal artery and the proper hepatic artery. As the proper hepatic artery travels toward the liver, it divides into the right hepatic and left hepatic arteries to supply the respective lobes of the liver [2,13]. The cystic artery (CA), which supplies the gallbladder, typically branches from the right hepatic artery (RHA) within the cystic triangle, a potential space bound by the cystic duct, common hepatic duct (CHD), and the inferior surface of the liver [14,15].

The second major unpaired branch of the abdominal aorta, the SMA, arises approximately 1-2 cm below the celiac trunk [16]. Typically, the first branch from the SMA is the inferior pancreaticoduodenal artery, which travels to the right and divides into the anterior and posterior inferior pancreaticoduodenal arteries. These vessels anastomose with the superior pancreaticoduodenal arteries (branching from the gastroduodenal artery) to supply the duodenum and pancreatic head [16]. Thus, the duodenum and pancreatic head receive arterial supplies from the celiac trunk and the SMA.

Clinical Significance of the Cystic Artery

The CA is a critical landmark for general surgeons performing laparoscopic cholecystectomies [11,15]. Careful dissection, identification, and ligation of the CA in the cystic triangle are imperative for the safe and efficacious removal of the gallbladder [15]. Notably, the CA has been noted to have variations in location, origin, and course. Therefore, a general surgeon needs a thorough understanding of the typical anatomy and potential variations of the CA to safely perform these procedures, reduce the number of iatrogenic injuries, and mitigate conversions into open procedures. The cystic triangle is an important anatomical and surgical landmark for identifying the CA [14,15]. The CA typically originates from the RHA within the cystic triangle before traveling laterally toward the gallbladder. The CA divides into a superficial and deep branch as it reaches the neck of the gallbladder. The superficial branch of the CA is responsible for supplying the free peritoneal surface. In contrast, the attached nonperitoneal surface of the gallbladder is supplied by the deep branch of the CA [14,15]. In cases where the CA or RHA have atypical origins that result in longer pathways to reach the gallbladder or liver, respectively, they will have spatial relationships with structures outside of the cystic triangle that they otherwise would not encounter. When a typical vascular pattern exists, the RHA is not a significant consideration for a pancreaticoduodenectomy, as this vessel is far from where the procedure occurs [7]. A Whipple procedure is used during this surgery to create cleavage planes in front of the SMA. As discussed below, one variation of RHA is that it can originate from SMA, making it a vessel surgeons must be aware of when performing a Whipple procedure to avoid iatrogenic injury [7].

The current work investigates variations in the cystohepatic blood supply of the right hepatic artery (RHA) and cystic artery (CA) in a midwestern American cadaveric sample. Knowledge of such variations may be beneficial for planning and safely performing surgical procedures involving the hepatobiliary system, as variant origins and paths change where these vessels are found and affect their spatial relationships with other structures.

## Materials and methods

All procedures in the current report were considered exempt by the A.T. Still University-Kirksville Institutional Review Board. Twenty-five white American Midwest cadavers from the Gift of Body Program at A.T. Still University's Kirksville College of Osteopathic Medicine were dissected to reveal the hepatobiliary anatomy. Exclusion criteria for selecting donor bodies were autopsy, donating major organs (except eyes), gross obesity, destructive trauma or extensive surgery, and communicable disease. A brief medical history was completed by the donor, or in the case of a next-of-kin donation, at the time of death. Information for the Missouri Certificate of Death was also required. Portal triads and associated vessels were carefully dissected to show the RHA and CA. In each dissection, the RHA's and CA's origin, branching pattern, and course were documented and photographed with a Canon EOS 6D Mark II camera. The length and midpoint diameter of these arteries were measured using electronic calipers, and summary statistics of these measurements were calculated using Microsoft Excel.

## Results

**Figure 1:**
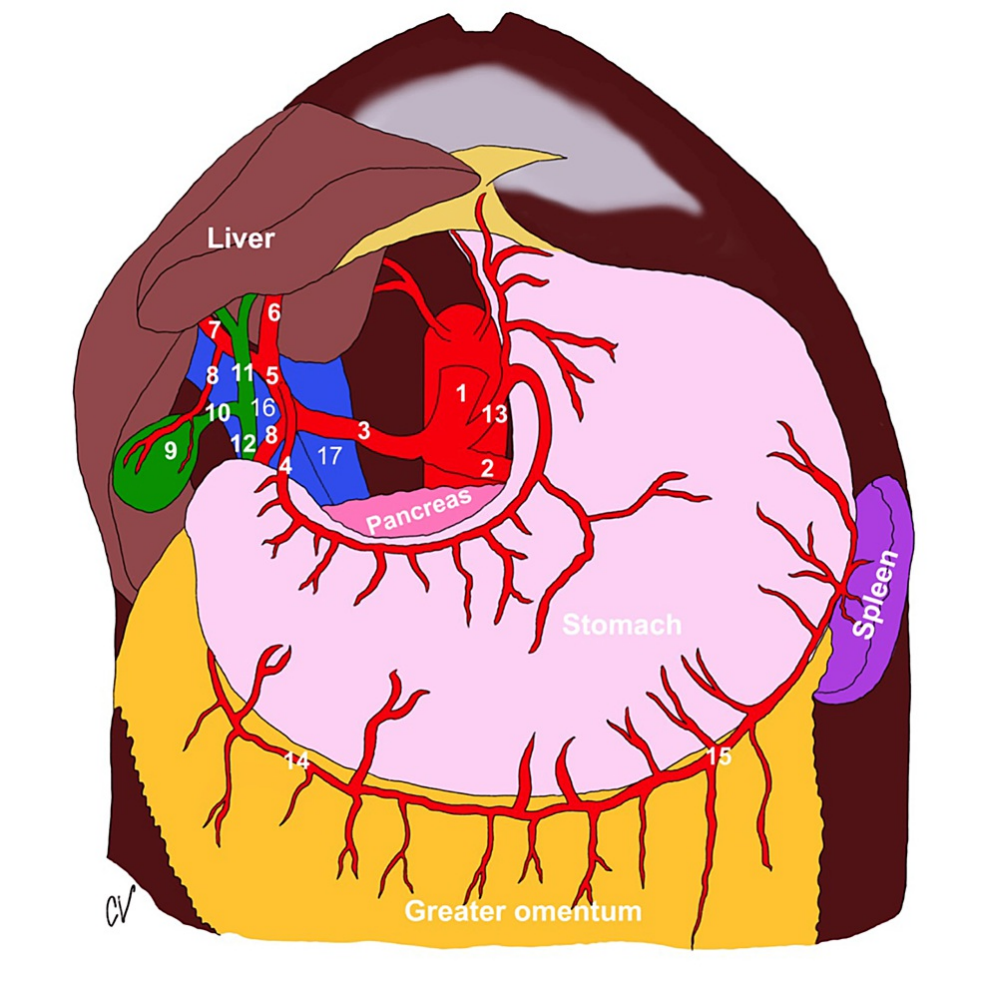
Celiac trunk anatomy 1. celiac trunk; 2. splenic artery; 3. common hepatic artery; 4. gastroduodenal artery; 5. proper hepatic artery; 6. left hepatic artery; 7. right hepatic artery; 8. cystic artery; 9. gall bladder; 10. cystic duct; 11. common hepatic duct; 12. common bile duct; 13. left gastric artery; 14. right gastroepiploic artery; 15. left gastroepiploic artery; 16. portal vein; 17. inferior vena cava (Image courtesy of Caroline VanSickle, Ph.D.)

The anatomy of the celiac trunk is depicted in Figure 1. 

Right Hepatic Artery Variations

Our findings included the four cases where the RHA originated from the anterolateral aspect of the proximal SMA (two males, two females, age range 50-90 years). These cases are described below, and measurements are summarized in Table 1.

**Table 1:** Measurements of RHA

	RHA length (mm)	RHA midpoint diameter (mm)
Case one: 79-year-old male	77.36	7.54
Case two: 89-year-old male	113.94	7.09
Case three: 90-year-old female	56.99	2.69
Case four: 54-year-old female	67.22	4.07
Mean of Cases 1-4	78.88 ± 24.81	5.35 ± 2.35

RHA Case One

**Figure 2:**
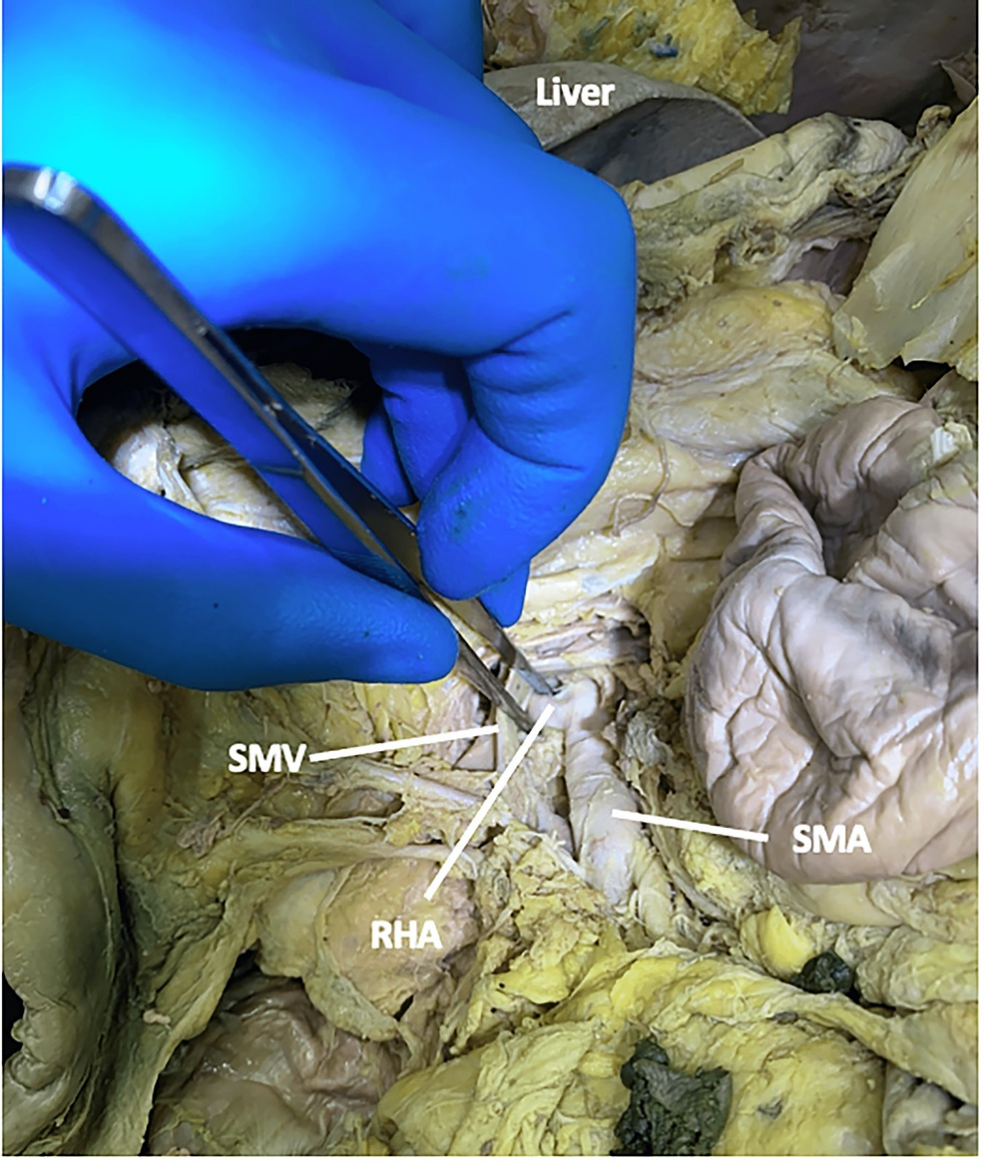
The origin of the right hepatic artery in case one Case one: In a 79-year-old male, the right hepatic artery originated from the superior mesenteric artery. RHA: right hepatic artery; SMA: superior mesenteric artery; SMV: superior mesenteric vein.

In this 79-year-old male, the RHA originated from the SMA (Figure 2).

**Figure 3:**
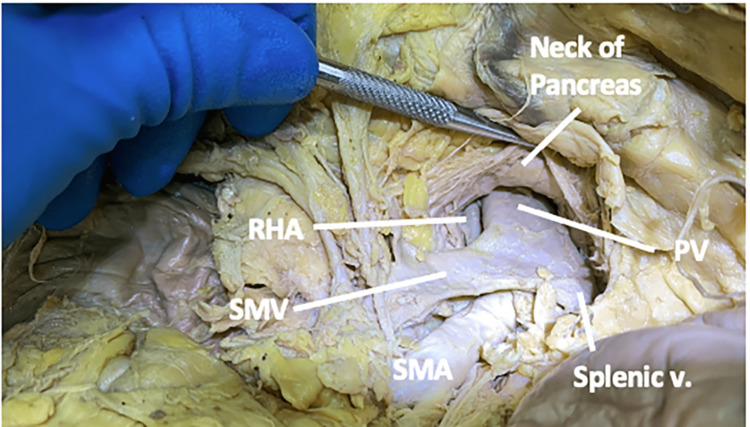
The spatial relationship of the right hepatic artery in case one In case one, the right hepatic artery traveled deep to the superior mesenteric vein and posterolaterally to the portal vein. PV: portal vein; RHA: right hepatic artery; SMA: superior mesenteric artery; SMV: superior mesenteric vein; v: vein.

The RHA traveled deep to the superior mesenteric vein and the pancreatic neck while running posterolaterally to the portal vein (Figure 3).

**Figure 4:**
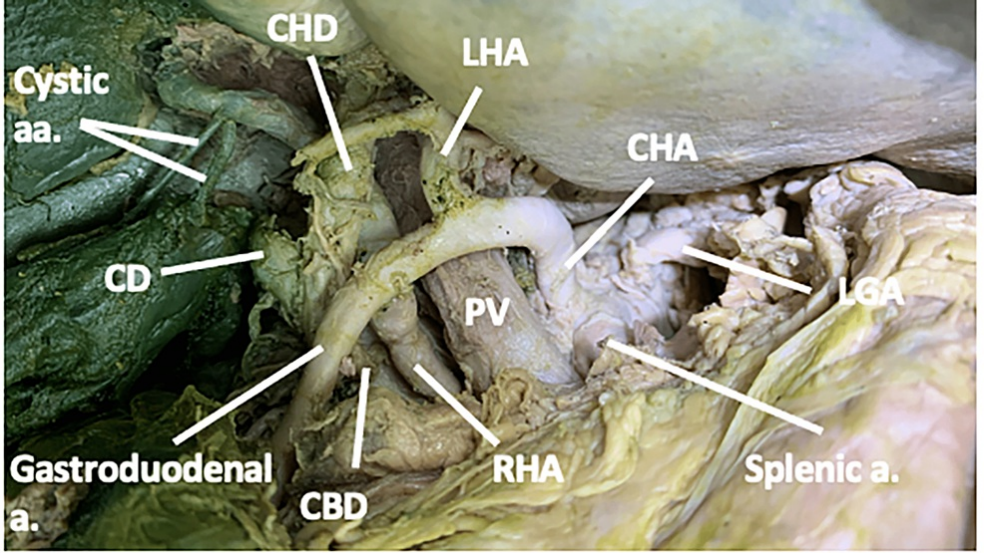
The spatial relationship between the right hepatic artery, the portal triad, and the cystic artery in case one In case one, the right hepatic artery passed deep to both the gastroduodenal artery and the common hepatic duct and gave off two cystic arteries between the portal vein and the common bile duct. a: artery; aa: arteries; CBD: common bile duct; CD: cystic duct; CHA: common hepatic artery; LGA: left gastric artery; LHA: left hepatic artery; CHD: common hepatic duct; PV: portal vein; RHA: right hepatic artery.

As the RHA approached the liver, it traveled between the portal vein and the common bile duct, passing deep to the gastroduodenal artery and the common hepatic duct (CHD). Within the cystic triangle, it gave off two cystic arteries before entering the liver (Figure 4).

RHA Case Two

**Figure 5:**
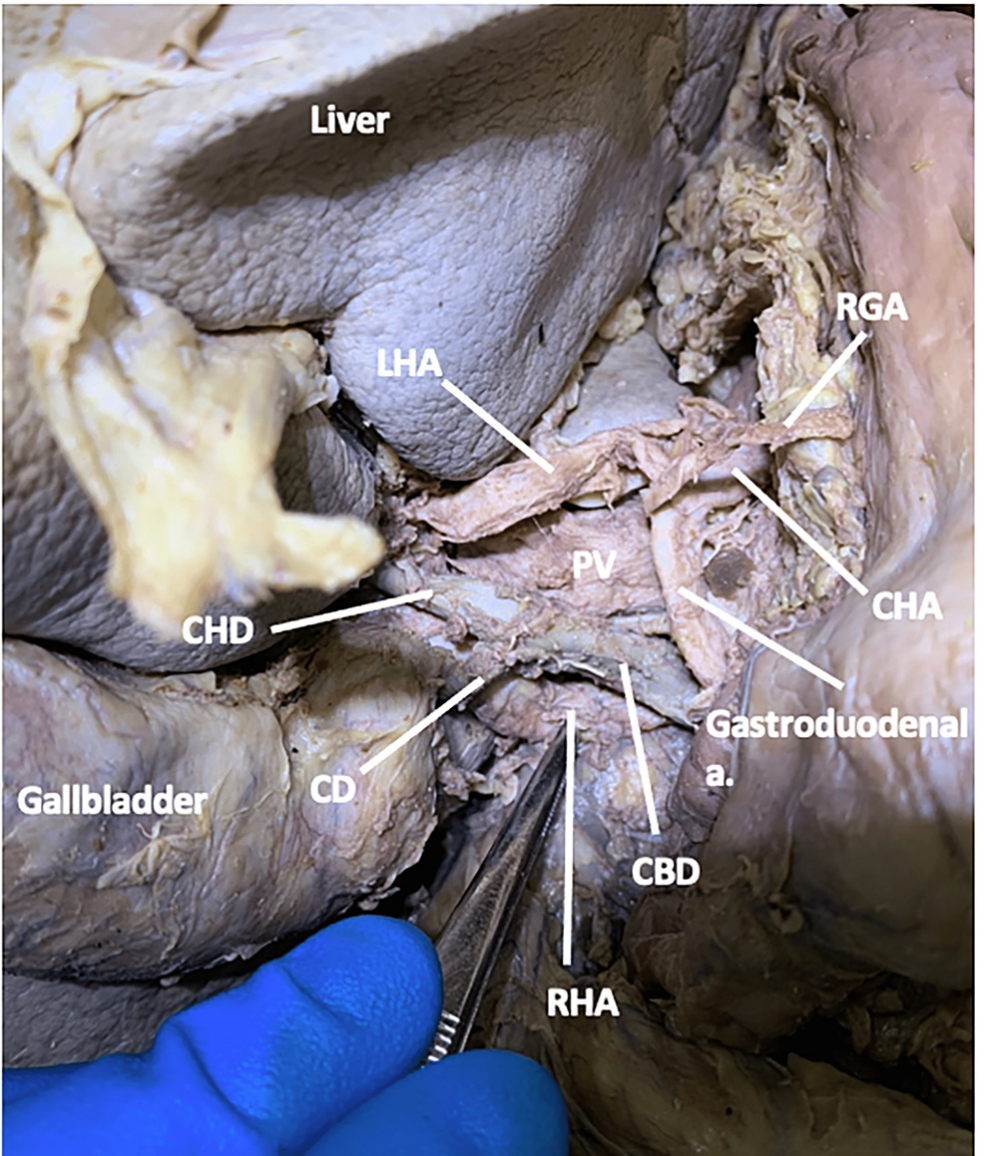
The spatial relationship between the right hepatic artery and the portal triad in case two Case two: In an 89-year-old male, the right hepatic artery originated from the superior mesenteric artery and traveled laterally to the portal vein, posterolaterally to the common bile duct, and deep to the cystic duct. a: artery; CBD: common bile duct; CD: cystic duct; CHA: common hepatic artery; LHA: left hepatic artery; CHD: common hepatic duct; PV: portal vein; RGA: right gastric artery; RHA: right hepatic artery

In this 89-year-old male, the RHA originated from the SMA and traveled deep to the super mesenteric vein and pancreatic neck. It continued laterally to the portal vein and posterolaterally to the common bile duct, eventually reaching the cystic duct (Figure 5).

**Figure 6:**
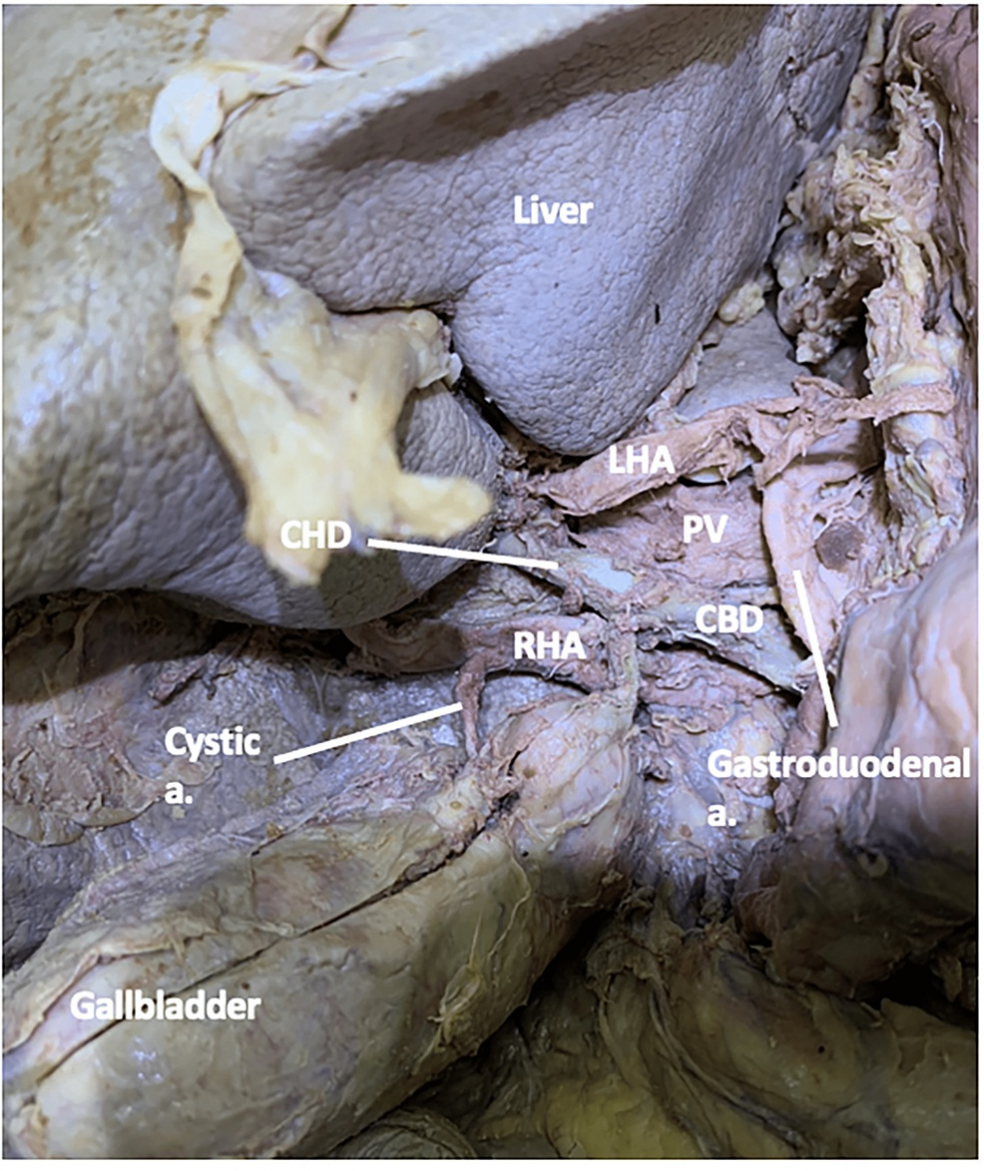
The spatial relationships between the right hepatic artery and the cystic triangle and the origin of the cystic artery in case two Case two: The cystic artery originated from the right hepatic artery in the cystic triangle. a: artery; CBD: common bile duct; LHA: left hepatic artery; CHD: common hepatic duct; PV: portal vein; RHA: right hepatic artery

Within the cystic triangle, the RHA gave off a single CA before entering the right lobe of the liver (Figure 6). 

RHA Case Three 

**Figure 7:**
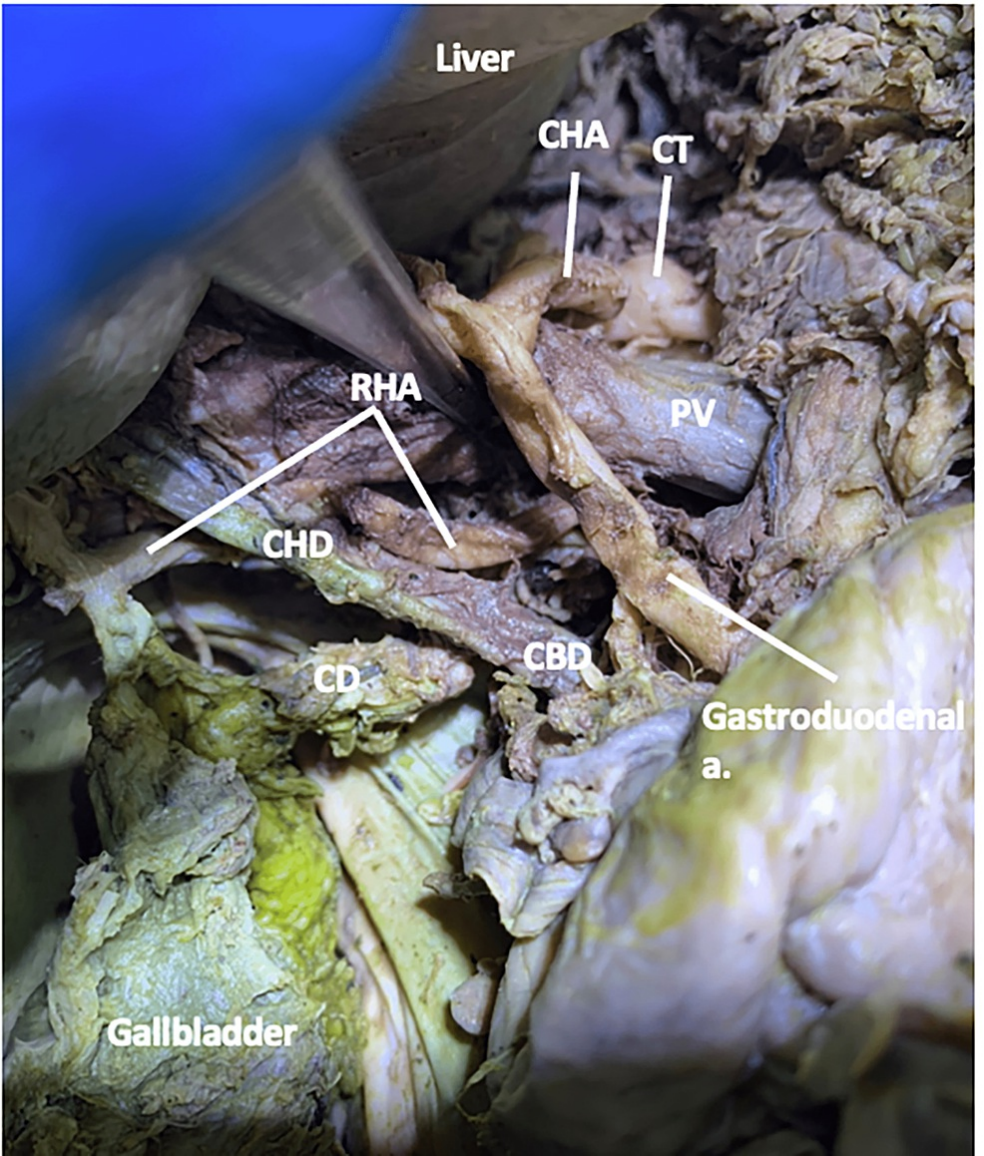
The spatial relationships of the right hepatic artery in case three Case 3: In a 90-year-old female, the right hepatic artery originated from the superior mesenteric artery and traveled deep to the pancreatic neck. The RHA coursed between the portal vein and the proper hepatic artery as it entered the cystic triangle. a: artery; CBD: common bile duct; CD: cystic duct; CHA: common hepatic artery; CT: celiac trunk; PHD: proper hepatic duct; PV: portal vein; RHA: right hepatic artery.

In this 90-year-old female, the RHA originated from the SMA, traveling deep to the pancreatic neck. The RHA traveled posterolaterally to the portal vein, moving even further laterally at the level of the formation of the common bile duct (Figure 7). The RHA coursed between the portal vein and the proper hepatic artery as it entered the cystic triangle (Figure 7).

**Figure 8:**
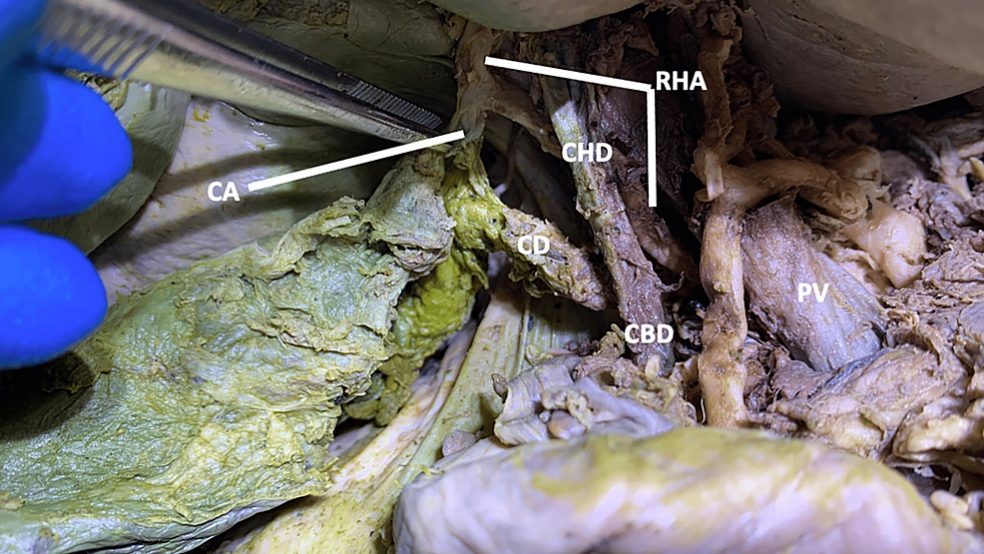
The spatial relationship of the right hepatic artery in the cystic triangle in case three Case three: The cystic artery from the right hepatic artery in the cystic triangle CA: cystic artery; CBD: common bile duct; CD: cystic duct; CHD: common hepatic duct; PV: portal vein; RHA: right hepatic artery

Within the cystic triangle, the RHA gave off a single CA before entering the right lobe of the liver (Figure 8).

RHA Case Four

**Figure 9:**
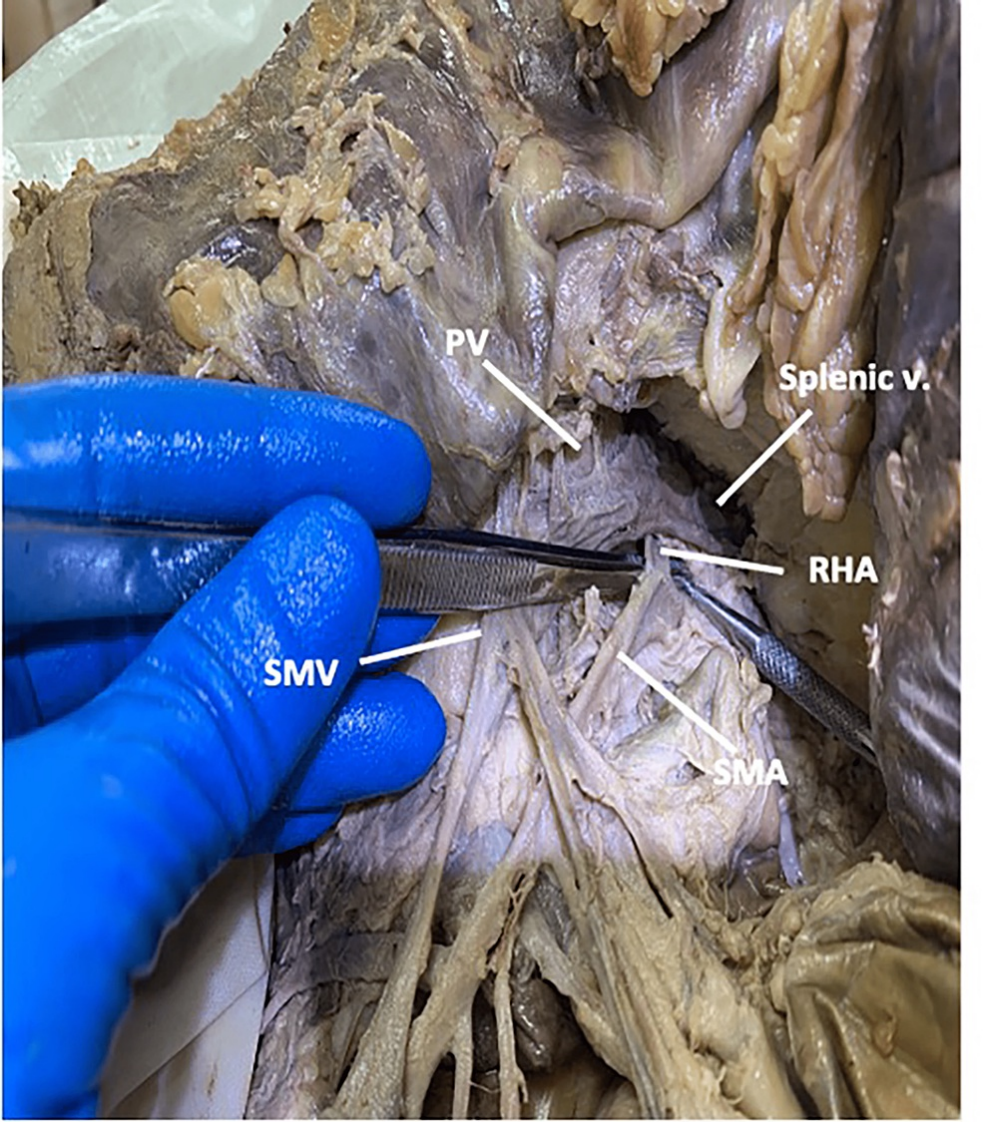
The origin and spatial relationships of the right hepatic artery in case four Case four: In a 54-year-old female, the right hepatic artery originated from the superior mesenteric artery and traveled deep to the distal portion of the splenic vein and the pancreatic neck. PV: portal vein; RHA: right hepatic artery; SMA: superior mesenteric artery; SMV: superior mesenteric vein; v: vein

In this 54-year-old female, the RHA originated from the SMA and traveled deep to the distal portion of the splenic vein and the pancreatic neck. The RHA traveled posterolaterally to the portal vein, gradually becoming more superficial superiorly (Figure 9).

**Figure 10:**
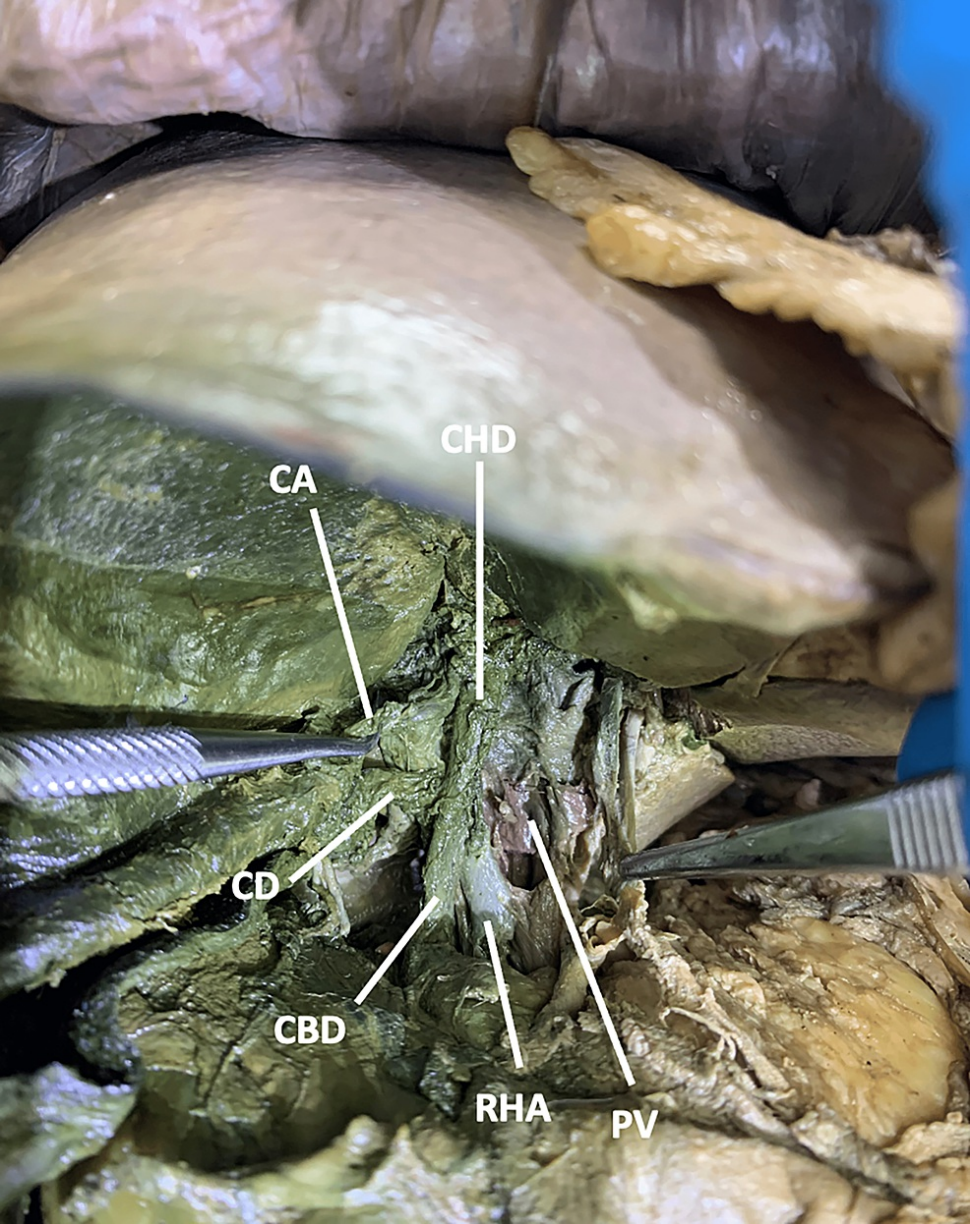
The spatial relationships between the right hepatic artery, the portal triad, and the cystic artery in case four Case four: The right hepatic artery traveled between the common bile duct and the portal vein and gave off a single cystic artery. CA: cystic artery; CBD: common bile duct; CD: cystic duct; CHD: common hepatic duct; PV: portal vein; RHA: right hepatic artery.

RHA continued between the common bile duct and the portal vein before taking a lateral trajectory deep into the bile system at the formation of the common bile duct (Figure 10).

Within the cystic triangle, the RHA gave off a single CA before entering the right lobe of the liver.

Cystic Artery Variations

**Figure 11:**
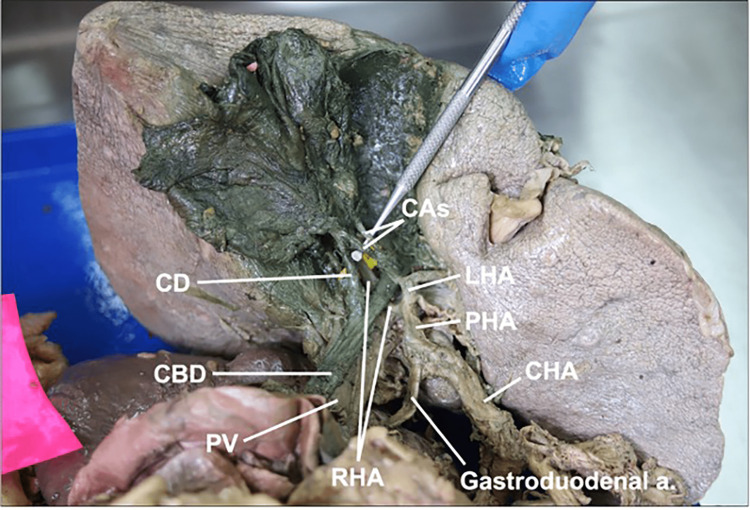
Cystic arteries arising from the left hepatic artery medial to the common bile duct a: artery; CAs: cystic arteries; CBD: common bile duct; CD: cystic duct; CHA: common hepatic artery; LHA: left hepatic artery; PHA: proper hepatic artery; PV: portal vein; RHA: right hepatic artery

Of 25 donor bodies, 18 individuals (11 males, seven females, mean age 72 ± 18 years) retained a gallbladder and were included in this study. The CA passed through the cystic triangle in all 18 cadavers. The mean length and midpoint diameter of the CA in all 18 individuals were 20.99 ± 5.95 mm and 1.59 ± 0.39 mm, respectively. We observed two types of CA variations relating to the origin of this vessel. First, we recorded which artery CA originated from. In our sample, the CA always originated from the RHA, except for one. In that case, two cystic arteries originated from the left hepatic artery and crossed superficially to the common hepatic duct to enter the cystic triangle, where they traveled superficially to the RHA to reach and supply the gallbladder (Figure 11). Second, we recorded whether or not the CA origin occurred within the cystic triangle. The CA originated within the cystic triangle in 12 individuals. In the six remaining cases, the CA originated outside of the cystic triangle, passing either deeply (n = 4) or superficially (n = 2) to the common hepatic duct before entering the cystic triangle to reach the gallbladder.

## Discussion

Anatomical variations of the abdominal vasculature, especially in the celiac trunk and SMA, are prevalent [1-4]. These variations impact diagnostics, interventions, and patient safety. Knowledge of potential variations and associated complications enhances diagnostic studies, preoperative planning, and the overall safety of surgical procedures. Therefore, familiarity with variations of the RHA and CA may have implications for laparoscopic surgical procedures of the hepatobiliary system (e.g., hemorrhage leading to conversion to an open procedure). In the current study, the RHA consistently supplied the right hepatic lobe and typically gave off the CA, which passed through the cystic triangle. In four cadavers, the RHA originated from the SMA instead of the proper hepatic artery. In one cadaver, the CA originated from the left hepatic artery.

Variation in the origin and course of RHA reported by previous researchers is similar to the results described here [9,17]. Michels et al. [17] analyzed 200 cadavers and found the frequency of hepatic artery variations to be 8%-11%; Balzan et al. [9] found the RHA variation frequency to be 13%-36%. In comparison, we found RHA variation in 22% (4 of 8 individuals) of our smaller sample, overlapping with the range reported by Balzan et al. [9]. Balzan et al. [9] reported on 12 cases where the RHA originated from SMA and took a similar path as it did in our cases, traveling deep to the pancreatic head and posterolaterally to the portal vein [9].

Understanding the origin and course of RHA is essential to avoiding severe complications during surgical procedures. During a laparoscopic cholecystectomy, improper ligation or iatrogenic injury to an abnormal RHA may result in hepatic infarction, liver abscesses, ischemic biliary injury, or an anastomotic fistula [7,9,10]. Such iatrogenic injuries may require an intraoperative conversion to an open cholecystectomy [18]. The Whipple procedure is used to create cleavage planes in front of the SMA. When the RHA originates from the SMA, it can complicate this step, increasing the risk of accidental damage. Similar concerns exist for other surgeries in this region that involve the RHA or nearby structures [7, 9,19].

The findings of the current study highlight the surgical importance of identifying hepatic arterial variations for proper planning and appropriate operative management of vascular encasement or arterial injury [9,10]. Except in cases of accessory vessels, simple ligation and section should be avoided because of the risk of hepatic necrosis and liver abscess [9,19]. Thus, surgeons and interventional radiologists must thoroughly understand the typical anatomy of the hepatic artery and its anatomic variations to avoid inadvertent injury when performing hepatobiliary surgeries, pancreatic surgeries, and arteriography in this region.

The frequency with which the CA originates from the RHA has previously been found to be 80% [14,15]; our study found a higher rate (94%, or 17 of 18 cadavers). Regarding variation in the path of the CA, the relationship between the CA and the biliary ducts is of particular concern for surgeons removing the gallbladder [11,15]. In the current study, one individual had a CA originating from the left hepatic artery instead of the RHA. In a cholecystectomy, unawareness of this variation may lead to hemorrhage and bile leakage, requiring conversion to an open cholecystectomy [5,6,8,11]. The CA can directly arise from other sites, including the left hepatic artery, common hepatic artery, proper hepatic artery, gastroduodenal artery, other celiac trunk derivatives, and even the SMA [5,8,11]. In our study, the CA originated from the RHA, even when the RHA originated from the SMA. This consistency may be due to the shared demography of our sample, as Singhal et al. [11] have suggested that certain variations are more common in some nationalities than others.

Limitations

The findings of this current study should be interpreted with caution. Our study examined a small and homogeneous sample, which limited our ability to consider how different factors affect the variation of these vessels.

## Conclusions

Within the small sample studied here, we found examples of variation in the origin and course of the RHA and CA. While the RHA typically originates from the proper hepatic artery and takes a short course to reach and supply the right lobe of the liver, we found four cases where the RHA originated from the SMA, traveling deep to the pancreatic head and posterolaterally to the portal vein. The typical origination does not lead to the RHA passing these structures. So typically, the RHA is not a consideration for surgical procedures (e.g., Whipple) involving the pancreatic head or surrounding structures. In one case, the CA originated from the left hepatic artery, crossing superficially to the common hepatic duct to enter the cystic duct and reach the gallbladder. When the CA originates from the RHA, as is typical, it does not usually cross the common hepatic duct. Evidence of variations in the origin and paths of the CA and RHA means that more surgeries require the surgeon to be aware of variations in these vessels to improve preoperative planning and the overall safety of such surgical procedures.
